# 5-Hydr­oxy-7-meth­oxy-4*H*-chromen-4-one

**DOI:** 10.1107/S1600536807066494

**Published:** 2007-12-18

**Authors:** Iván Brito, Jorge Bórquez, Luis Alberto Loyola, Matías López-Rodríguez

**Affiliations:** a Departamento de Química, Facultad de Ciencias Básicas, Universidad de Antofagasta, Casilla 170, Antofagasta, Chile; b Instituto de Bio-Orgánica ‘Antonio González’, Universidad de La Laguna, Astrofísico Francisco Sánchez No. 2, La Laguna, Tenerife, Spain

## Abstract

The mol­ecular conformation of the title compound, C_10_H_8_O_4_, isolated from *Laretia acualis*, is stabilized by a strong intra­molecular hydrogen bond between the hydroxyl and carbonyl groups. The crystal packing shows π–π stacking inter­actions. The chromene (4*H*-1-benzopyran-4-one) unit is essentially planar.

## Related literature

For related literature, see: Gabor (1988 ▶); Valenti *et al.* (1993 ▶, 1998 ▶); Vasconcelos *et al.* (1998 ▶); Bernstein *et al.* (1995 ▶); Wickens (1995 ▶); Wallet & Cody (1995 ▶).
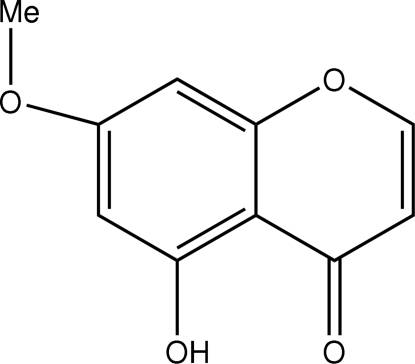


## Experimental

### 

#### Crystal data

C_10_H_8_O_4_
*M* *_r_* = 192.16
Monoclinic, 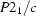

*a* = 9.7551 (3) Å
*b* = 11.7512 (9) Å
*c* = 7.5211 (7) Å
β = 95.094 (4)°
*V* = 858.77 (11) Å^3^
*Z* = 4
Mo *K*α radiation
μ = 0.12 mm^−1^
*T* = 298 (2) K
0.19 × 0.10 × 0.08 mm

#### Data collection

Nonius KappaCCD area-detector diffractometer
Absorption correction: none
1504 measured reflections
1504 independent reflections
1393 reflections with *I* > 2σ(*I*)

#### Refinement

*R*[*F* ^2^ > 2σ(*F* ^2^)] = 0.041
*wR*(*F* ^2^) = 0.115
*S* = 1.08
1504 reflections
131 parameters
H atoms treated by a mixture of independent and constrained refinement
Δρ_max_ = 0.15 e Å^−3^
Δρ_min_ = −0.17 e Å^−3^

### 

Data collection: *COLLECT* (Nonius, 1998 ▶); cell refinement: *DENZO–SMN* (Otwinowski & Minor, 1997 ▶); data reduction: *DENZO–SMN*; program(s) used to solve structure: *SIR97* (Altomare *et al.*, 1999 ▶); program(s) used to refine structure: *SHELXL97* (Sheldrick, 1997 ▶); molecular graphics: *ORTEP-3 for Windows* (Farrugia, 1997 ▶); software used to prepare material for publication: *WinGX* (Farrugia, 1999 ▶).

## Supplementary Material

Crystal structure: contains datablocks global, I. DOI: 10.1107/S1600536807066494/bt2661sup1.cif

Structure factors: contains datablocks I. DOI: 10.1107/S1600536807066494/bt2661Isup2.hkl

Additional supplementary materials:  crystallographic information; 3D view; checkCIF report

## Figures and Tables

**Table 1:** Hydrogen-bond geometry (Å, °)

*D*—H⋯*A*	*D*—H	H⋯*A*	*D*⋯*A*	*D*—H⋯*A*
O3—H9⋯O2	0.92 (3)	1.72 (3)	2.5901 (17)	155 (2)

**Table 2:** π–π interactions (Å,°) *Cg*1 and *Cg*2 are the centroids of rings O1/C2–C4/C4*A*–C8*A* and C4*A*/C5–C8/C8*A*, respectively. The offset is defined as the distance between *CgI* and the perpendicular projection of *CgJ* on ring *I*.

*CgI*	*CgJ*	*CgI*⋯*CgJ*	Dihedral angle	Interplanar distance	Offset
*Cg*1	*Cg*2^i^	3.6661 (8)	1.39	3.51	1.13
*Cg*2	*Cg*1^ii^	3.6660 (8)	1.39	3.49	1.06
*Cg*2	*Cg*2^i^	3.7930 (8)	1.69	3.47	1.50
*Cg*2	*Cg*2^ii^	3.7931 (8)	1.69	3.49	1.53

Symmetry codes: (i) 
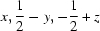
; (ii) 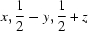.

**Fig. 1.**
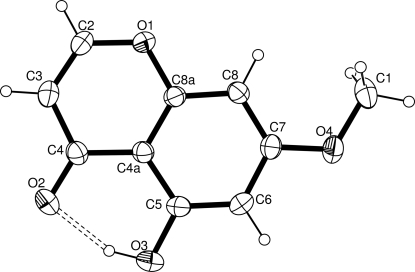
The molecular structure of the title compound, showing the atom-numbering scheme. Displacement ellipsoids are draw at the 30% probability level. The dashed line indicates the intramolecular O–H···O hydrogen bond. H atoms are shown as small spheres of arbitrary radii.

## supplementary crystallographic information

### Comment

The title compound was originally isolated from Artemisia campestris (maritima) (Vasconcelos *et al.*, 1998) and later from Laretia acualis (Cav.) which is known in Chile as "Llareta de la zona central", is a yellowish-green, compact resinous cushion shrub, which grows in the high Andes of Chile. Whole plant infusions are widely used as diabetes treatment in folk medicine (Wickens, 1995). Chromene derivatives exhibits a wide spectrum of biological activity, including spasmolytic, anti-arrhytmic, cardionthonic, antiviral, anticancer and alkylating properties (Gabor, 1988; Valenti *et al.*, 1993, 1998).

The title structure (Fig.1), consists of a chromene moiety substituted in position 5 and 7 with a hydroxy and methoxy group, respectively. The chromene ring system is essentially planar with maximum deviation of -0.007 (2) Å for C2. The geometrical parameters of the chromene group are comparable to those of related structures reported earlier (Wallet & Cody, 1995). The mean bond distances are: O-C*sp*^2 ^1.3552 (17) Å, and aromatic C—C 1.391 (2) Å, while C4=O2 is 1.2531 (18)Å and C2=C3 is essentially a double bond with a distance of 1.330 (2) Å Å. In the crystal structure, the molecular packing is stabilized by intramolecular O—H···O hydrogen bond generating a graph-set motif S(6) (Bernstein *et al.*, 1995) as well as π- π stacking interactions (Table 2).

### Experimental

Dried and finely powdered tissues from the aerial parts of *Laretia acualis* (535 g) were extracted with petrol ether at room temperature. The solvent was evaporated to dryness by vacuum distillation and low temperature, yielding a gum (15 g). The concentrated petrol ether extract was fractionated on silica gel column with hexane-ethyl acetate mixtures of increasing polarity as elution solvents. The fraction hexane-ethyl acetate 10% (2.45 g) was separated on silica gel using the same elution solvents yielding 45.5 mg of (I)(m.p. 374 K). The title compound was identified by comparing the spectroscopic data with the previously published data (Vasconcelos *et al.*, 1998). Recrystallization from hexane/ethyl acetate (8:2) at room temperature afforded colourless crystals suitable for X-ray diffraction analysis.

### Refinement

H atom attached to O3 atom was located in a difference Fourier map and refined isotropically. All other H atoms were positioned geometrically and then treated as riding, with C—H distances of 0.93 (CH) and 0.96 Å (CH_3_), and *U*_iso_(H) = 1.2*U*_eq_(C) or *U*_iso_(H) = 1.5*U*_eq_(C_methyl_).

### Crystal data

**Table:**

C_10_H_8_O_4_	*F*_000_ = 400
*M**_r_* = 192.16	*D*_x_ = 1.486 Mg m^−^^3^
Monoclinic, *P*2_1_/*c*	Melting point: 374 K
Hall symbol: -P 2ybc	Mo *K*α radiation λ = 0.71073 Å
*a* = 9.7551 (3) Å	Cell parameters from 1504 reflections
*b* = 11.7512 (9) Å	θ = 3.0–25.0º
*c* = 7.5211 (7) Å	µ = 0.12 mm^−^^1^
β = 95.094 (4)º	*T* = 298 (2) K
*V* = 858.77 (11) Å^3^	Prismatic, colourless
*Z* = 4	0.19 × 0.10 × 0.08 mm

### Data collection

**Table:**

Nonius KappaCCD area-detector diffractometer	1393 reflections with *I* > 2σ(*I*)
Radiation source: fine-focus sealed tube	*R*_int_ = 0
Monochromator: graphite	θ_max_ = 25.3º
φ scans, and ω scans with κ offsets	θ_min_ = 3.2º
Absorption correction: none	*h* = −11→11
1504 measured reflections	*k* = −14→0
1504 independent reflections	*l* = 0→9

### Refinement

**Table:**

Refinement on *F*^2^	H atoms treated by a mixture of independent and constrained refinement
Least-squares matrix: full	*w* = 1/[σ^2^(*F*_o_^2^) + (0.06*P*)^2^ + 0.1521*P*] where *P* = (*F*_o_^2^ + 2*F*_c_^2^)/3
*R*[*F*^2^ > 2σ(*F*^2^)] = 0.041	(Δ/σ)_max_ < 0.001
*wR*(*F*^2^) = 0.115	Δρ_max_ = 0.15 e Å^−^^3^
*S* = 1.08	Δρ_min_ = −0.17 e Å^−^^3^
1504 reflections	Extinction correction: none
131 parameters	

### Special details

**Table:**

Geometry. All e.s.d.'s (except the e.s.d. in the dihedral angle between two l.s. planes) are estimated using the full covariance matrix. The cell e.s.d.'s are taken into account individually in the estimation of e.s.d.'s in distances, angles and torsion angles; correlations between e.s.d.'s in cell parameters are only used when they are defined by crystal symmetry. An approximate (isotropic) treatment of cell e.s.d.'s is used for estimating e.s.d.'s involving l.s. planes.
Refinement. Refinement of *F*^2^ against ALL reflections. The weighted *R*-factor *wR* and goodness of fit *S* are based on *F*^2^, conventional *R*-factors *R* are based on *F*, with *F* set to zero for negative *F*^2^. The threshold expression of *F*^2^ > σ(*F*^2^) is used only for calculating *R*-factors(gt) *etc*. and is not relevant to the choice of reflections for refinement. *R*-factors based on *F*^2^ are statistically about twice as large as those based on *F*, and *R*- factors based on ALL data will be even larger.

### Fractional atomic coordinates and isotropic or equivalent isotropic displacement parameters (Å^2^)

**Table:**

	*x*	*y*	*z*	*U*_iso_*/*U*_eq_	
O1	0.70907 (10)	0.05272 (8)	0.30079 (14)	0.0533 (3)	
O2	0.96609 (11)	0.31131 (10)	0.18700 (17)	0.0649 (4)	
O3	0.77689 (13)	0.45485 (9)	0.25771 (16)	0.0629 (4)	
H9	0.855 (3)	0.422 (2)	0.219 (3)	0.098 (7)*	
O4	0.36201 (10)	0.31332 (9)	0.43512 (14)	0.0558 (3)	
C1	0.27108 (16)	0.22189 (16)	0.4674 (2)	0.0633 (5)	
H1A	0.1856	0.2521	0.5006	0.095*	
H1B	0.3121	0.175	0.5622	0.095*	
H1C	0.2544	0.177	0.3609	0.095*	
C2	0.83521 (16)	0.03322 (13)	0.2464 (2)	0.0586 (4)	
H2	0.8626	−0.042	0.235	0.07*	
C3	0.92292 (15)	0.11442 (14)	0.2080 (2)	0.0568 (4)	
H3	1.0084	0.0946	0.1715	0.068*	
C4	0.88700 (14)	0.23206 (12)	0.22245 (19)	0.0460 (4)	
C4A	0.75193 (13)	0.25315 (10)	0.27872 (16)	0.0384 (3)	
C5	0.69920 (14)	0.36468 (11)	0.29717 (17)	0.0427 (3)	
C6	0.57034 (15)	0.38106 (12)	0.35134 (17)	0.0460 (4)	
H6	0.5372	0.4545	0.3645	0.055*	
C7	0.48861 (13)	0.28748 (12)	0.38688 (16)	0.0419 (3)	
C8	0.53533 (13)	0.17729 (11)	0.37016 (17)	0.0419 (3)	
H8	0.4808	0.1152	0.394	0.05*	
C8A	0.66641 (13)	0.16281 (11)	0.31662 (17)	0.0391 (3)	

### Atomic displacement parameters (Å^2^)

**Table:**

	*U*^11^	*U*^22^	*U*^33^	*U*^12^	*U*^13^	*U*^23^
O1	0.0439 (6)	0.0339 (5)	0.0831 (7)	0.0016 (4)	0.0111 (5)	−0.0009 (4)
O2	0.0439 (6)	0.0602 (7)	0.0922 (8)	−0.0118 (5)	0.0150 (5)	0.0044 (6)
O3	0.0628 (8)	0.0378 (6)	0.0895 (8)	−0.0086 (5)	0.0146 (6)	0.0063 (5)
O4	0.0431 (6)	0.0615 (7)	0.0646 (6)	0.0096 (5)	0.0145 (5)	−0.0015 (5)
C1	0.0409 (8)	0.0829 (12)	0.0677 (10)	−0.0004 (8)	0.0138 (7)	0.0061 (8)
C2	0.0456 (8)	0.0432 (8)	0.0876 (11)	0.0098 (6)	0.0105 (7)	−0.0038 (7)
C3	0.0376 (8)	0.0554 (9)	0.0779 (10)	0.0068 (7)	0.0087 (7)	−0.0033 (7)
C4	0.0357 (7)	0.0483 (8)	0.0536 (8)	−0.0036 (6)	0.0015 (5)	0.0015 (6)
C4A	0.0353 (7)	0.0387 (7)	0.0405 (6)	−0.0017 (5)	−0.0012 (5)	0.0006 (5)
C5	0.0458 (8)	0.0357 (7)	0.0458 (7)	−0.0036 (6)	0.0001 (5)	0.0023 (5)
C6	0.0501 (8)	0.0372 (7)	0.0502 (7)	0.0085 (6)	0.0026 (6)	−0.0011 (5)
C7	0.0376 (7)	0.0504 (8)	0.0374 (6)	0.0040 (6)	0.0020 (5)	−0.0012 (5)
C8	0.0380 (7)	0.0408 (7)	0.0470 (7)	−0.0039 (5)	0.0043 (5)	0.0015 (5)
C8A	0.0377 (7)	0.0347 (7)	0.0443 (7)	0.0007 (5)	0.0002 (5)	−0.0007 (5)

### Geometric parameters (Å, °)

**Table:**

O1—C2	1.3503 (18)	C3—C4	1.433 (2)
O1—C8A	1.3674 (15)	C3—H3	0.93
O2—C4	1.2531 (17)	C4—C4A	1.4407 (19)
O3—C5	1.3510 (16)	C4A—C8A	1.3952 (18)
O3—H9	0.92 (2)	C4A—C5	1.4192 (18)
O4—C7	1.3522 (16)	C5—C6	1.369 (2)
O4—C1	1.428 (2)	C6—C7	1.398 (2)
C1—H1A	0.96	C6—H6	0.93
C1—H1B	0.96	C7—C8	1.3821 (19)
C1—H1C	0.96	C8—C8A	1.3848 (18)
C2—C3	1.330 (2)	C8—H8	0.93
C2—H2	0.93		
			
C2—O1—C8A	118.65 (11)	C8A—C4A—C5	117.02 (12)
C5—O3—H9	103.8 (15)	C8A—C4A—C4	120.54 (12)
C7—O4—C1	118.20 (12)	C5—C4A—C4	122.44 (12)
O4—C1—H1A	109.5	O3—C5—C6	120.20 (12)
O4—C1—H1B	109.5	O3—C5—C4A	119.18 (13)
H1A—C1—H1B	109.5	C6—C5—C4A	120.62 (12)
O4—C1—H1C	109.5	C5—C6—C7	120.04 (12)
H1A—C1—H1C	109.5	C5—C6—H6	120
H1B—C1—H1C	109.5	C7—C6—H6	120
C3—C2—O1	124.40 (14)	O4—C7—C8	123.45 (13)
C3—C2—H2	117.8	O4—C7—C6	115.13 (12)
O1—C2—H2	117.8	C8—C7—C6	121.42 (12)
C2—C3—C4	120.62 (14)	C7—C8—C8A	117.52 (12)
C2—C3—H3	119.7	C7—C8—H8	121.2
C4—C3—H3	119.7	C8A—C8—H8	121.2
O2—C4—C3	122.79 (13)	O1—C8A—C8	115.95 (11)
O2—C4—C4A	122.08 (13)	O1—C8A—C4A	120.66 (12)
C3—C4—C4A	115.12 (12)	C8—C8A—C4A	123.38 (12)
			
C8A—O1—C2—C3	0.7 (2)	C1—O4—C7—C8	−1.15 (18)
O1—C2—C3—C4	−0.1 (3)	C1—O4—C7—C6	177.84 (12)
C2—C3—C4—O2	−179.70 (15)	C5—C6—C7—O4	−178.42 (11)
C2—C3—C4—C4A	−0.5 (2)	C5—C6—C7—C8	0.6 (2)
O2—C4—C4A—C8A	179.81 (12)	O4—C7—C8—C8A	178.87 (11)
C3—C4—C4A—C8A	0.59 (19)	C6—C7—C8—C8A	−0.05 (19)
O2—C4—C4A—C5	0.2 (2)	C2—O1—C8A—C8	179.00 (12)
C3—C4—C4A—C5	−178.99 (12)	C2—O1—C8A—C4A	−0.52 (19)
C8A—C4A—C5—O3	−178.38 (11)	C7—C8—C8A—O1	−179.75 (11)
C4—C4A—C5—O3	1.2 (2)	C7—C8—C8A—C4A	−0.24 (19)
C8A—C4A—C5—C6	0.52 (19)	C5—C4A—C8A—O1	179.50 (10)
C4—C4A—C5—C6	−179.88 (11)	C4—C4A—C8A—O1	−0.11 (19)
O3—C5—C6—C7	178.07 (11)	C5—C4A—C8A—C8	0.02 (19)
C4A—C5—C6—C7	−0.8 (2)	C4—C4A—C8A—C8	−179.59 (11)

### Hydrogen-bond geometry (Å, °)

**Table:**

*D*—H···*A*	*D*—H	H···*A*	*D*···*A*	*D*—H···*A*
O3—H9···O2	0.92 (3)	1.72 (3)	2.5901 (17)	155 (2)

### Table 2 π–π interactions (Å,°)

**Table:**

CgI	CgJ	CgI···CgJ	Dihedral angle	Interplanar distance	Offset
Cg1	Cg2^i^	3.6661 (8)	1.39	3.508	1.13
Cg2	Cg1^ii^	3.6660 (8)	1.39	3.487	1.06
Cg2	Cg2^i^	3.7930 (8)	1.69	3.471	1.50
Cg2	Cg2^ii^	3.7931 (8)	1.69	3.486	1.53

Symmetry codes: (i)x, 1/2-y, -1/2+z; (ii) x, 1/2-y, 1/2+z. Cg1 and Cg2 are the centroids of rings O1/C2-C4/C4A–C8A and C4A/C5–C8/C8A, respectively. The offset is defined as the distance between CgI and the perpendicular projection of CgJ on ring I.
